# Risk Factors for Noninitiation and Dropout in Blended Therapy in Inpatient Psychiatric Patients: Retrospective Cohort Study

**DOI:** 10.2196/85816

**Published:** 2026-06-29

**Authors:** Nikita Gupta, Fabian Gardin, Thomas Berger, Wolfram Kawohl

**Affiliations:** 1 Clienia AG Oetwil am See Switzerland; 2 University of Bern Bern Switzerland; 3 University of Zurich Zurich Switzerland; 4 University of Nicosia Nicosia Cyprus

**Keywords:** blended therapy, noninitiation, dropout, inpatients, psychiatric, predictors

## Abstract

**Background:**

Blended therapy (BT) combines digital applications with face-to-face treatment and has become an increasingly important component of psychiatric care. Evidence indicates that BT can achieve outcomes comparable to or even superior to those of traditional face-to-face therapy. Despite certain advantages, routine implementation of BT remains challenging, and clinical practice suggests that while some inpatients engage with BT, many either discontinue early or do not initiate its use at all. To better understand these patterns, this multicentric, retrospective observational study investigates factors associated with noninitiation and dropout among inpatients who are offered BT.

**Objective:**

In this study, data from 278 inpatients were analyzed to examine the influence of sociodemographic variables, comorbidities, and symptom severity on the uptake and continued use of BT. The objective was to identify predictors of noninitiation and dropout.

**Methods:**

Multivariable logistic regression models were conducted to identify significant predictors of noninitiation and dropout among inpatients using the transdiagnostic, cognitive behavioral therapy–based electronic mental health platform Minddistrict, which offers modules targeting psychoeducation, cognitive restructuring, and behavioral activation. Data were collected from 2 psychiatric hospitals between January 2020 and May 2024. The sample consisted predominantly of patients diagnosed with depression (182/278, 65.7%) and posttraumatic stress disorder (61/278, 21.9%), alongside various comorbid conditions.

**Results:**

The findings indicate distinct patterns of association for noninitiation and dropout. Of the 278 patients, only 5 (1.8%) completed all the assigned modules, and one-third of the patients never initiated the platform at all. Specifically, increasing age was linked to a lower risk of noninitiation (odds ratio [per year age difference] 0.98, 95% CI 0.96-1.00; *P*=.01), while the presence of a comorbid anxiety disorder was associated with a reduced risk of dropout (odds ratio 0.23, 95% CI 0.08-0.66; *P*=.007). Several variables showed no association with either noninitiation or dropout across all analyses, including sex, overall symptom severity, and certain comorbidities such as personality disorders and depression.

**Conclusions:**

In this preselected inpatient sample, uptake of BT was very limited. Older age was associated with lower noninitiation, and comorbid anxiety disorders were associated with a lower likelihood of dropout. These findings may help inform future prospective studies on how BT can be introduced and supported more effectively in inpatient psychiatric care. As access to BT was granted selectively by therapists, the results should be interpreted as predictors of engagement within a selected sample rather than general predictors of BT uptake among all psychiatric inpatients.

## Introduction

Blended therapy (BT) is defined as the combination of digital applications, such as computer-based or internet-based programs, with face-to-face therapy. This combination of therapy modalities can be implemented in various forms. A substantial body of literature indicates that BT can achieve outcomes comparable or even superior to those of traditional standalone face-to-face therapy [1,2].

Despite its numerous advantages, implementing BT in routine practice remains challenging. This is due to various factors, including the high resource demands associated with the use of BT [3,4].

Online interventions are often characterized by high dropout and low adherence rates [5,6], highlighting the need to systematically investigate the underlying mechanisms behind it, as adherence can be a relevant factor for therapeutic success in prior research on treatment outcomes [7,8]. Understanding these factors is essential for building a comprehensive knowledge of how to optimize and implement this BT effectively. Evidence suggests that dropout is not a random phenomenon but is influenced by specific factors, particularly sociodemographic characteristics and symptom severity [9,10]. For example, Wu et al [10] examined predictors of noninitiation and dropout in blended cognitive behavioral therapy and found that both sociodemographic and clinical variables, such as depression severity and early digital engagement, significantly influenced adherence. Similarly, a meta-analysis by Karyotaki et al [9] and earlier findings by Christensen et al [11] confirmed that younger age, limited knowledge about psychological treatments, and greater symptom severity are associated with lower adherence in internet-based interventions. It should be noted that there is limited scientific literature specifically addressing BT, as most of the existing literature focuses on the area of unguided and guided self-help. Nonetheless, it is reasonable to assume that the use of BT also involves diverse patient-specific and diagnosis-specific influencing factors.

In the real-life implementation of BT at the psychiatric hospital *Clienia Schlössli AG*, many patients were offered blended treatment, but only a few actually started and completed the modules. This showed a relevant gap between initial setup and active participation in the treatment. This discrepancy is noteworthy, especially because a prior qualitative study conducted at the same hospital suggested that patients who engaged with BT were generally satisfied with its implementation [4]. This may indicate that acceptability among users does not necessarily translate into broad uptake or sustained use in routine inpatient care. Identifying risk factors for a low uptake of BT and/or low adherence could be therefore crucial to optimize the selection of suitable patients for this therapeutic approach. Adherence, defined as the completion of assigned modules on the platform, is associated with better treatment outcomes for depression [12]. In contrast, metrics such as the number of log ins or the total time spent on the platform have shown no correlation with improved outcomes [13]. Therefore, this study emphasizes module completion as a key indicator of engagement and its potential association with improved therapeutic outcomes. A distinctive feature of this study is its examination of therapy noninitiation, a phenomenon of clinical relevance that is exemplified by the case discussed earlier. While most studies on adherence primarily address dropout, referring to participants who begin but do not complete therapy, patients who never appropriately start therapy are often excluded from analysis. This exclusion may result in the loss of valuable information about a potentially important subgroup of patients.

A more targeted and personalized application of BT in selected patient groups may promote more efficient use of clinical resources. This is particularly relevant given that previous studies have reported that the implementation of BT can require additional time, effort, and resources [3,4,14]. Therefore, a part of optimizing resources should be the careful selection of patients who can truly benefit from its application.

The population examined in this study consists of patients receiving inpatient treatment. This is particularly relevant because the evidence base for BT in inpatient settings remains limited. Although only a few studies have examined its use in this context, the implementation of BT in inpatient care represents a valuable extension of this therapeutic approach.

The primary objective is to examine potential links between the noninitiation and dropout of BT and various influencing factors, building on findings from previous studies investigating similar phenomena.

## Methods

### BT Intervention

The study is a multicentric, retrospective observational study. We analyzed data from patients who used the electronic mental health platform *Minddistrict* at 2 psychiatric hospitals during their inpatient treatment. *Minddistrict* is a platform founded in the Netherlands in 2008, and it provides a transdiagnostic catalog structured into modules, self-help training, and diaries, drawing on cognitive behavioral therapy principles. The platform’s modules primarily target psychoeducation, cognitive restructuring, and behavioral activation. It allows for individualized treatment by assigning suitable interventions tailored to each patient’s needs. Patients can monitor behavioral patterns and track therapy progress through diaries or independently select interventions from the self-help catalog. *Minddistrict* additionally offers various communication channels, including video calls and chat functions. Access to *Minddistrict* was granted to selected and interested patients by their therapists, who created user accounts on their behalf. After the account creation, patients could engage with the platform through therapist-guided modules, which required therapists to unlock new content after certain steps were completed. Additionally, patients had the option to use diaries, where they could document and reflect on their experiences independently and without therapist involvement. *Minddistrict* additionally offers various communication channels, including video calls and chat functions. The use of the video calls and diary features was excluded from this study because these components do not involve structured BT interventions.

### Inclusion and Exclusion Criteria

The analysis includes inpatients from 2 psychiatric hospitals: *Clienia Schlössli* and *Clienia Littenheid*, which accessed the electronic mental health platform between January 2020 and May 2024. Platform use was reassessed in August 2024 to capture a comprehensive overview of module engagement and log in frequency. Although it is possible that some patients completed additional modules or increased use after this cutoff, such cases are considered unlikely. Patients were excluded if no modules had been assigned by their therapists. Patient accounts with multiple log ins and without module assignments were excluded, as we assumed that the platform was only used for video calls rather than for structured BT in these cases.

### Division of Patient Groups

As outlined in the Introduction section, this study investigates noninitiation, defined as the failure to begin the intervention, and dropout, defined as the failure to complete any of the assigned therapeutic modules after platform initiation. To enable this analysis, patients were categorized into the following groups:

- Group A: patients who never logged in or only logged in once while not completing any modules
- Group B: patients who logged in once and completed one or more modules
- Dropout: patients who completed none of their assigned modules
- Nondropout: patients who completed at least one of their assigned modules

**Figure 1:**
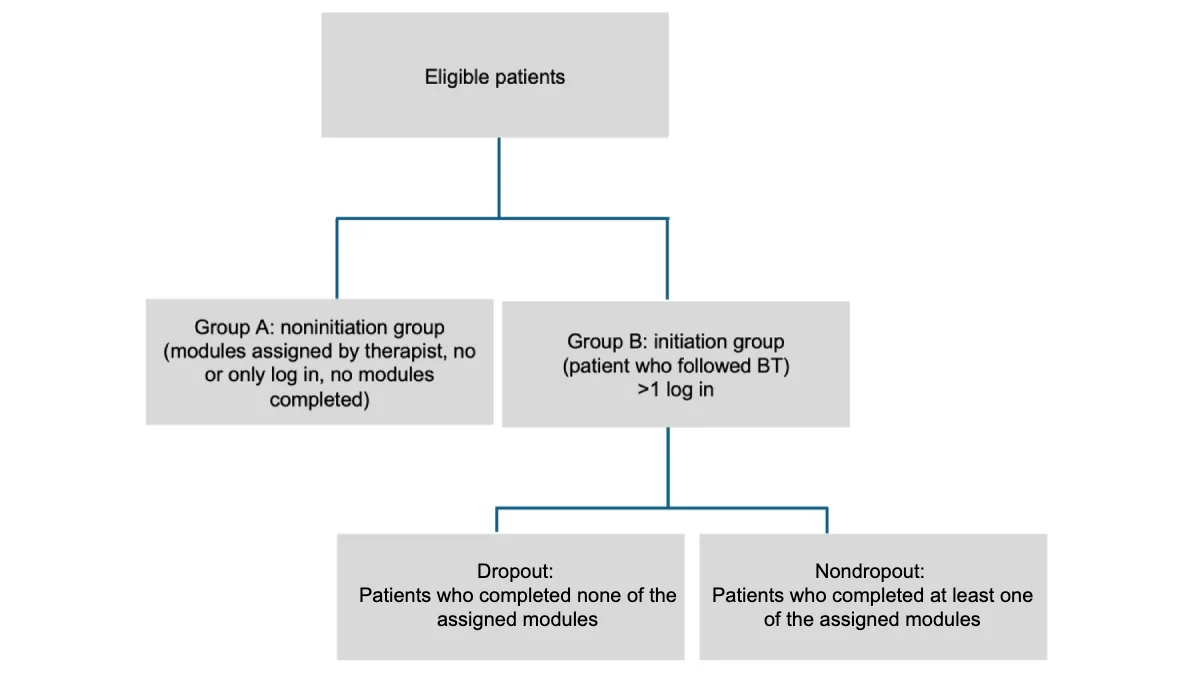
Flowchart of patient classification into noninitiation and initiation groups. BT: blended therapy.

A schematic overview of the patient categorization process is presented in Figure 1.

### Outcome Measures

#### Overview

The data sources for this study included electronic medical records containing routinely collected datasets at the time of patient admission. Additionally, data on user activity on the platform are obtained directly from the *Minddistrict*’s database. An overview of all collected data is presented in Textbox 1.

**Overview of the collected data.**

**Sociodemographic characteristics**

- Age
- Gender
- Marital status
- Educational level
- Employment status
- Setting (inpatient vs outpatient)

**Assessments for symptom severity**

- Brief Symptom Checklist
- Health of the Nation Outcome Scales

**Diagnosis**

- Main diagnosis
- Comorbid diagnoses

**Platform use**

- Number of log ins to the platform
- Number of modules assigned by the therapist
- Number of modules completed by the patient

#### Assessment of Symptom Severity

##### Brief Symptom Checklist

The Brief Symptom Inventory is a standardized self-report questionnaire used as a psychometric instrument to assess psychological distress. It consists of 53 items rated on a 5-point Likert scale and covers 9 symptom dimensions, including somatization, obsessiveness, interpersonal insecurity, depressiveness, anxiety, aggressiveness or hostility, phobic anxiety, paranoid thinking, and psychoticism. The Brief Symptom Checklist (BSCL) is the licensed German adaptation of the Brief Symptom Inventory. In the clinical setting, assessment is based on the total score of the BSCL, which was documented at admission and discharge. This allows the evaluation of symptom change over the course of inpatient treatment. Internal consistency (Cronbach α) ranged from satisfactory to very good across subscales, with the highest values for depressiveness and the Global Severity Index. Test-retest reliability after 1 week has been satisfactory to good across most dimensions [15].

##### Health of the Nation Outcome Scales

The Health of the Nation Outcome Scales (HoNOS) is a clinician-rated scale used to assess 12 items covering behavior, impairment, symptoms, and social functioning, including work and daily life. Each item is rated from 0 to 4, with a maximum score of 48, with higher scores indicating higher problem burden. The scale is routinely completed at hospital admission and discharge to track symptom changes. HoNOS is considered a practical tool for assessing the severity of mental health problems in inpatient settings. Existing literature supports its good interrater and test-retest reliability [16].

### Ethical Considerations

This study (project ID: 2024-00768) has been reviewed and approved by the Zurich Ethics Committee. This research was conducted as a retrospective cohort study; therefore, active informed consent was not required by the ethics committee. Patient data were pseudonymized prior to analysis. Identifying information was stored separately from the analytic dataset and accessible only to authorized study personnel.

### Data Analysis

Descriptive statistics include means and SDs for continuous variables, as well as numbers and percentages for categorical variables. Multivariable logistic regression models were used to identify factors associated with the extent of platform tool use based on a predefined set of variables, including age, sex, education, employment, civil status, disease severity (HoNOS or BSCL), treatment setting, and main diagnosis. Missing values were handled using multiple imputation by chained equations with the *mice* package in R [17]. Appropriate imputation methods were applied depending on the variable type, including logistic, polytomous, proportional odds regressions, and predictive mean matching. A total of 70 datasets were imputed for the whole dataset with 30 iterations, and 45 datasets were imputed for the stationary setting subset with 15 iterations. All analyses were carried out with R (version 4.4.3; R Foundation for Statistical Computing), and RMarkdown (Posit Software) was used for dynamic reporting.

## Results

### Descriptive Analysis

#### Distribution of Patient Characteristics

Descriptive statistics for the full inpatient sample (N=278) are presented in Table 1. The mean age was 45.45 (SD 15.14) years, and the sample consisted of 112 (40.3%) patients who were male. Regarding civil status, most patients were either single (96/236, 40.7%) or married or cohabiting (92/236, 38.9%). In terms of educational background, the largest proportion had completed an apprenticeship or vocational school (114/263, 43.3%), followed by university or higher education (54/263, 20.5%). Employment status varied considerably, with 94 (34.4%) out of 273 patients unemployed, 67 (24.5%) patients employed full-time, and 60 (21.9%) patients receiving disability or retirement benefits.

Most patients were diagnosed with depression (182/278, 65.7%) or posttraumatic stress disorder (61/278, 21.9%). Comorbidity was common, with a mean of 2.56 (SD 1.34) diagnoses per patient. The most frequent comorbid conditions were posttraumatic stress disorder (82/278, 29.6%), substance use disorder (65/278, 23.5%), and personality disorder (55/278, 19.9%).

Disease severity decreased substantially over the course of inpatient treatment. Mean HoNOS scores improved from 18.04 (SD 4.54) at admission to 9.22 (SD 4.42) at discharge, and BSCL scores decreased from 78.36 (SD 33.57) to 42.79 (SD 31.64).

**Table 1:** Descriptive characteristics of the patient sample (N=278).

Characteristics		Values	Missing
Sex (male), n (%)		112 (40.3)	0 (0)
Age (years), mean (SD)		45.45 (15.14)	0 (0)
**Civil status, n (%)**			42 (15.1)
	Single	96 (40.7)	
	In a registered partnership	1 (0.4)	
	Married or cohabiting	92 (39)	
	Divorced	44 (18.6)	
	Widowed	3 (1.3)	
**Educational level, n (%)**			15 (5.4)
	Not completed school education	4 (1.5)	
	Compulsory school education	41 (15.6)	
	Apprenticeship or full-time vocational school	114 (43.3)	
	Higher vocational school or technical college	46 (17.5)	
	Secondary school (Matura level)	4 (1.5)	
	University or higher education institution	54 (20.5)	
**Employment status, n (%)**			5 (1.8)
	Full-time employed	67 (24.5)	
	Part-time employed	52 (19)	
	Unemployed or not employed	94 (34.4)	
	Disability insurance (IV)^a^, old-age pension (AHV)^b^, or another pension form	60 (22)	
**Primary diagnosis, n (%)**			1 (0.4)
	Depression	182 (65.7)	
	Bipolar disorders	9 (3.2)	
	Personality disorder	8 (2.9)	
	Pain-related disorder and somatoform disorder	1 (0.4)	
	Posttraumatic stress disorder	61 (22)	
	Anxiety or fear-related disorders	4 (1.4)	
	Eating disorder	2 (0.7)	
	Adaptation disorder	6 (2.2)	
	Obsessive-compulsive disorder	0 (0)	
	Schizophrenia or other primary psychotic disorders	1 (0.4)	
	Disorders due to substance use	2 (0.7)	
	Other	1 (0.4)	
**Comorbidity**			
	Number of diagnoses, mean (SD)	2.56 (1.34)	0 (0)
	Depression, n (%)	250 (90.3)	1 (0.4)
	Personality disorder, n (%)	55 (19.9)	1 (0.4)
	Substance use disorder, n (%)	65 (23.5)	1 (0.4)
	Posttraumatic stress disorder, n (%)	82 (29.6)	1 (0.4)
	Anxiety disorder, n (%)	36 (13)	1 (0.4)

^a^IV: Invalidenversicherung (disability insurance).

^b^AHV: Alters- und Hinterlassenenversicherung (national old-age pension scheme).

#### Distribution of Platform Use Parameters

On average, patients logged into the system 5.9 (SD 14.4; median 2.0, IQR 1.0-5.8) times. The mean number of modules assigned was 3.8 (SD 3.4; median 3.0, IQR 1.0-5.0), while the mean number of completed modules was 0.9 (SD 2.0; median 0.0, IQR 0.0-1.0), corresponding to a mean module completion rate of 24% (0.9/3.8). Notably, only 5 (1.8%) out of 278 patients completed all assigned modules. In terms of group distribution, 94 (33.8%) patients were in group A (noninitiation group), and 184 (66.2%) patients initiated the platform. Among those who initiated the platform, 94 (51.1%) completed none of their assigned modules (dropout), while 90 (48.9%) completed at least one assigned module (nondropout).

### Regression Models With Imputed Data

#### Logistic Regression Model on Factors Potentially Associated With Noninitiation

In the logistic regression model assessing factors associated with noninitiation of the online platform among inpatients (N=278), age was the only variable significantly associated with noninitiation. Specifically, increasing age was linked to a lower likelihood of noninitiation (odds ratio [OR]=0.98, 95% CI 0.96-1.00; *P*=.01). While the OR is presented as 1, the significant *P* value indicates an actual effect, with the actual OR likely being marginally below 1 but not captured due to rounding. The odds decreased by 2% for each 1-year increase in age, suggesting that older patients were more likely to engage with the platform. For an age difference of 10 years, this would indicate an OR of 0.8. All other variables were not significantly associated with noninitiation (Table 2).

**Table 2:** Logistic regression model of factors potentially associated with noninitiation.

Parameter	Odds ratio (95% CI)	*P* value
Sex (male)	1.05 (0.60-1.84)	.86
Age (difference per year)	0.98 (0.96-1.00)	.01
Depression	1.24 (0.51-3.03)	.63
Personality disorder	0.68 (0.34-1.37)	.28
Substance use disorder	1.34 (0.73-2.46)	.35
Posttraumatic stress disorder	1.22 (0.66-2.26)	.52
Anxiety disorder	1.12 (0.52-2.40)	.77
HoNOS^a^ entry	0.98 (0.93-1.05)	.59
BSCL^b^ entry	1.04 (0.94-1.15)	.41

^a^HoNOS: Health of the Nation Outcome Scales.

^b^BSCL: Brief Symptom Checklist.

#### Logistic Regression Model on Factors Potentially Associated With Dropout

In the logistic regression model assessing factors associated with dropout, anxiety disorder emerged as a statistically significant predictor. Patients diagnosed with an anxiety disorder had significantly lower odds of dropping out (OR 0.23, 95% CI 0.08-0.66; *P*=.007), indicating a higher likelihood of continued engagement with the platform. In addition, 2 nonsignificant trends were observed. First, patients with a personality disorder showed a tendency toward lower dropout rates (OR 0.50, 95% CI 0.21-1.19; *P*=.11), suggesting a potential association that may need further investigation. Second, there was a weak trend indicating that higher symptom severity at admission, as measured by the HoNOS score, might be associated with increased dropout risk (OR 1.07, 95% CI 0.99-1.15; *P*=.08), though this finding did not reach statistical significance (Table 3).

**Table 3:** Logistic regression model of factors potentially associated with the dropout of the online platform.

Parameter	Odds ratio (95% CI)	*P* value
Sex (male)	0.88 (0.44-1.74)	.70
Age (difference per year)	0.98 (0.96-1.01)	.15
Depression	1.1 (0.36-3.31)	.86
Personality disorder	0.5 (0.21-1.19)	.11
Substance use disorder	1.25 (0.57-2.73)	.57
Posttraumatic stress disorder	0.88 (0.40-1.95)	.74
Anxiety disorder	0.23 (0.08-0.66)	.007
HoNOS^a^ entry	1.07 (0.99-1.15)	.08
BSCL^b^ entry	1.05 (0.93-1.19)	.39

^a^HoNOS: Health of the Nation Outcome Scales.

^b^BSCL: Brief Symptom Checklist.

## Discussion

### Summary and Interpretation of Findings

This study identified 2 significant predictors of engagement with BT among inpatients: increasing age was associated with a lower risk of noninitiation, while comorbid anxiety disorder was associated with a lower risk of dropout. These findings are particularly noteworthy as they differ depending on whether noninitiation or dropout is examined, and these associations were observed in a heterogeneous patient population encompassing a broader range of diagnoses than that included in a previous study [10], which was limited to patients with anxiety disorders and depression. In contrast, our study examines a more heterogeneous patient population with a wider range of diagnoses, allowing a more comprehensive analysis of the BT’s applicability across different conditions. The statistical analysis reveals significant findings, which interestingly differ depending on whether we examine the issue of noninitiation or dropout.

Previous studies have demonstrated that nonparticipation in general psychotherapy does not occur at random over time. The risk is particularly high at the beginning of the therapeutic process, often even before the first therapy session [18]. This finding is consistent with our study, where approximately one-third of the participants (94/278, 33.8%) were classified as belonging to the noninitiation group. This pattern suggests that not initiating a therapy may be driven not by the intervention itself but by other underlying factors. Therefore, it is essential to monitor this vulnerable phase closely and provide enhanced support precisely at this critical phase.

A striking finding of this study is the overall low uptake of the intervention. Despite being offered to a preselected group of interested patients, only 5 (1.8%) out of 278 patients completed all the assigned modules. One-third of patients never initiated the platform at all, and of those who did, most did not complete their assigned modules, resulting in a mean module completion rate of approximately 24% (mean 0.9, SD 2.0 out of 3.8, SD 3.4 assigned modules). These findings are consistent with the broader literature on digital interventions, which has repeatedly documented high rates of nonuse and early disengagement [5,6]. Several factors may help explain this low overall uptake. First, the absence of standardized follow-up procedures likely played a relevant role. Without systematic monitoring of patient engagement, low levels of platform use may have gone unnoticed or unaddressed by therapists [4]. Second, the lack of standardized criteria for module assignment may have contributed to low completion rates. Therapists in a qualitative study conducted at the same institution reported uncertainty about which modules were suitable for which patients and emphasized that active integration and follow-up are essential for meaningful engagement and that module assignment alone is insufficient unless supported by structured guidance [4].

Furthermore, the findings suggest that increasing age has a protective effect against noninitiation, indicating that older patients may be more likely to begin the intervention after modules have been assigned by the therapist. Previous research investigating internet-based cognitive behavioral therapy has shown that older adults tend to complete more modules and remain engaged over longer periods compared to younger users [19-22]. These studies suggest that older individuals may benefit from greater persistence, show more stable use patterns, and show potentially higher motivation, resulting in better adherence and lower dropout rates in digital interventions. One hypothesis could be that older individuals may adhere slightly better to digital interventions because they are more likely to be retired and therefore may have more time available to engage with therapy. However, this explanation does not apply to this study, as all participants were treated under inpatient conditions and therefore had access to comparable time resources. This suggests that factors other than time availability may underlie the higher adherence observed among older patients. A possible explanation is that younger users may approach digital interventions in a more exploratory way while lacking a strong initial commitment to the intervention. If immediate benefits are not apparent, they may disengage quickly. In contrast, older adults may begin treatment with more realistic expectations and greater persistence. Their decision to engage with digital therapy, despite potential barriers such as unfamiliarity with technology or initial skepticism, may be due to a high baseline level of intrinsic motivation. Younger individuals, for whom digital tools are more embedded in daily life, may face lower entry thresholds, resulting in a more heterogeneous group with varying levels of motivation, including those with only low therapeutic interest. Additionally, older adults who agree to participate in digital treatment may possess other characteristics associated with treatment success, such as higher education, more cognitive resources, or greater health-related self-efficacy.

Our findings indicate that patients with comorbid anxiety disorders in inpatient settings have a lower likelihood of discontinuing therapy. Anxiety disorders frequently cooccur with other psychiatric conditions, making it important to investigate their potential influence on the chance of dropouts. The prior evidence regarding the relationship between comorbid anxiety and psychotherapy dropout has been mixed and inconsistent. Studies have examined anxiety as a predictor of dropout both in general psychotherapy settings and specifically in the context of internet-based interventions. Some studies suggest that comorbid anxiety is associated with lower dropout rates [23], while others report the opposite, indicating a higher likelihood of early termination [24]. The evidence remains inconclusive. This raises the question of why patients with anxiety disorders in our study were less likely to drop out. One possible hypothesis relates to personality characteristics. Evidence suggests that individuals with anxiety disorders more frequently display cluster C personality traits [25]. These traits are associated with high conscientiousness, and a strong sense of duty can have a link to improved treatment adherence. Such individuals may be more willing to follow therapeutic guidance and continue engaging with assigned modules due to their conscientious traits. Another possible explanation for the lower dropout rate among patients with comorbid anxiety is that the structured and predictable nature of BT aligns well with their needs. The clear framework and guidance of online modules may support adherence by complementing traits commonly associated with anxiety. Additionally, the platform’s transdiagnostic content may target essential mechanisms of anxiety, such as rumination and avoidance, which may increase the perceived personal relevance of the material. In addition, anxious patients also tend to closely monitor their own progress, which may support ongoing participation, particularly in digital formats that involve self-tracking or structured exercises. It is also possible that individuals with anxiety disorders require additional effort to engage in social situations, including those encountered during face-to-face psychotherapy. psychotherapy. Working on a digital platform may reduce the potentially distressing effects of direct interpersonal interaction, which could make digital therapy a more appealing and manageable option for them. Finally, the higher frequency of contact points in BT may lead to a sense of safety and continuity that helps reduce uncertainty.

### Implications of the Findings

The results of this study could lead to 2 possible approaches. On the one hand, it might be beneficial to provide additional support to patients with a high risk of dropout or noninitiation to ensure successful completion of the therapy, for instance, by giving special attention to these patients. On the other hand, one could argue that these patients may not benefit from this therapy form and that alternative therapeutic approaches should be prioritized for them. Considering the wide range of available therapy options, certain approaches may simply be better suited for specific conditions. Wentzer et al [26] aimed to develop a tool to systematically assess factors critical for successful outcomes in BT, focusing on aspects such as computer access, internet skills, acute crises, and patient motivation. Similarly, Karyotaki et al [27] applied a personalized medicine approach in a systematic review to improve therapy allocation for patients with depression. Using sociodemographic characteristics and baseline symptom severity as predictors, the authors developed an interactive decision-support tool to guide treatment allocation among guided digital therapy, unguided digital therapy, and treatment as usual. While these studies concentrated on either practical barriers or depression-specific treatment optimization, this study extends this approach by including a broader patient population across multiple diagnoses. Such efforts are essential to optimize both treatment outcomes and resource use. In the future, standardized tools could help predict dropout risk, thereby enabling more targeted and effective implementation of BT. Accurate identification of suitable patients may enhance therapeutic efficiency and reduce health care costs. However, the findings of this study should be interpreted within their specific context. Our analysis focuses exclusively on adherence to BT, without assessing treatment outcomes. It is conceivable that some patients completed modules without having any clinical benefit, for example, to meet expectations from their therapists. Nevertheless, previous research has shown that homework adherence is positively associated with therapeutic success [28]. In line with this, we assume that module completion in BT also contributes to improved outcomes. By identifying risk factors for noninitiation and dropout, this study offers valuable insights into an underexplored area of digital mental health. It supports a more personalized and cost-efficient approach to psychiatric care by helping to match BT more precisely to patients who are likely to benefit. It expands the existing evidence base and contributes to the advancement of targeted, evidence-based treatment strategies.

### Limitations

Our findings must be interpreted considering several limitations. A limitation is the heterogeneity of the data, collected over an extended period (January 2020 to May 2024), with considerable variability in therapist training, application methods, and patient selection. No standardized procedures were applied, and information on selection criteria is missing. Importantly, as access to the Minddistrict platform was granted selectively by therapists to interested patients, no systematic data were collected on patients who were not offered BT during the study period. This precludes any formal comparison between included and nonincluded patients, and the extent and direction of the resulting selection bias therefore remains unknown. Future prospective studies should consider collecting data on all potentially eligible patients, regardless of whether they are ultimately offered BT, to allow for such comparisons.

Moreover, therapist-related variables are incomplete. Prior research shows that the extent and consistency of guidance significantly affect adherence [29,30] and that therapists’ attitudes toward digital tools play a role in patient engagement. The therapeutic relationship is also a known protective factor against dropout [31]. The absence of standardized training and varying application strategies may have contributed to differences in adherence. Additional influencing factors such as self-efficacy, expectations, digital affinity, and therapeutic alliance were not assessed. Finally, while the study reveals associations between predictors and dropout or noninitiation, it does not clarify underlying mechanisms. Qualitative studies could offer further insights, and future prospective studies with standardized protocols and more homogeneous samples are recommended. These limitations should be considered when interpreting the findings.

## Abbreviations

BSCL: Brief Symptom Checklist
BT: blended therapy
HoNOS: Health of the Nation Outcome Scales
OR: odds ratio

## Data Availability

The datasets generated or analyzed during this study are available from the corresponding author on reasonable request.
