# Correction: Risk Factors for Noninitiation and Dropout in Blended Therapy in Inpatient Psychiatric Patients: Retrospective Cohort Study

**DOI:** 10.2196/111245

**Published:** 2026-10-22

**Authors:** Nikita Gupta, Fabian Gardin, Thomas Berger, Wolfram Kawohl

**Affiliations:** 1 Clienia AG Oetwil am See Switzerland; 2 University of Bern Bern Switzerland; 3 University of Zurich Zurich Switzerland; 4 University of Nicosia Nicosia Cyprus

In “Risk Factors for Noninitiation and Dropout in Blended Therapy in Inpatient Psychiatric Patients: Retrospective Cohort Study” [1], the group definitions underlying the dropout analysis were described incorrectly. The Methods section and Figure 1 state that the dropout comparison was defined as “Group B1: patients who completed 100% of all assigned modules” versus “Group B2: patients who completed <100% of all assigned modules.” However, the logistic regression model reported in Table 3 was in fact conducted using a different definition: patients who completed none of their assigned modules (dropout) versus patients who completed at least one assigned module (nondropout).

All statistical analyses and results reported in the article (Tables 2 and 3, including all odds ratios, CIs, and *P* values) were calculated using this correct definition and remain valid and unchanged. The error is limited to the description of the group definitions in the Methods section, Figure 1, and corresponding wording in the Results and Discussion.

The interpretation of the dropout-related findings is refined by the correction: associations reflect the likelihood of completing no assigned modules at all, rather than the likelihood of incomplete module completion, as could be inferred from the original text. The noninitiation analysis is unaffected by this correction.

First, in the Division of Patient Groups section of the Methods, the following statement was revised:

Group B1: patients who completed 100% of all assigned modules

Group B2: patients who completed <100% of all assigned modules

The corrected text now reads:

Dropout: patients who completed none of their assigned modules

Nondropout: patients who completed at least one of their assigned modules

Second, in the same section, the following statement was revised:

...and dropout, the incomplete use of the assigned therapeutic modules.

The corrected text now reads:

...and dropout, defined as the failure to complete any of the assigned therapeutic modules after platform initiation.

Third, in Figure 1, the lower-level box labels were revised from the following:

Group B1: Patients who completed 100% of the assigned modules

Group B2: Patients who completed <100% of the assigned modules

The corrected figure now reads:

Dropout: Patients who completed none of the assigned modules

Nondropout: Patients who completed at least one of the assigned modules

Fourth, in the Distribution of Platform Use Parameters section of the Results, the following sentence was revised:

Notably, only 5 (1.8%) out of 278 patients completed all assigned modules (group B1).

The corrected text now reads:

Notably, only 5 (1.8%) out of 278 patients completed all assigned modules.

Fifth, in the same section, the following sentence was revised:

In terms of group distribution, 94 (33.8%) patients were in group A (noninitiation group), 184 (66.2%) patients initiated the platform, of whom 179 (64.4%) patients did not complete all assigned modules (group B2).

The corrected text now reads:

In terms of group distribution, 94 (33.8%) patients were in group A (noninitiation group), and 184 (66.2%) patients initiated the platform. Among those who initiated the platform, 94 (51.1%) completed none of their assigned modules (dropout), while 90 (48.9%) completed at least one assigned module (nondropout).

Finally, in the Discussion section, the following statement was revised:

...more willing to follow therapeutic guidance and complete assigned modules due to their conscientious traits.

The corrected text now reads:

...more willing to follow therapeutic guidance and continue engaging with assigned modules due to their conscientious traits.

The correction will appear in the online version of the paper on the JMIR Publications website together with the publication of this correction notice. Because this was made after submission to PubMed, PubMed Central, and other full-text repositories, the corrected article has also been resubmitted to those repositories.
